# Aquatic-derived antimicrobial peptides and their strategically modified analogues as prospective anticancer therapeutics: a comprehensive systematic review of enhancement methodologies and mechanistic insights

**DOI:** 10.3389/fchem.2026.1876776

**Published:** 2026-07-07

**Authors:** Jacob Wekalao, Tobias Topisia

**Affiliations:** 1 School of Engineering and Technology, National Forensic Sciences University, Gujarat, India; 2 Department of Physics, School of Pure and Applied Sciences, Maasai Mara University, Narok, Kenya

**Keywords:** anticancer peptides, antimicrobial peptides, apoptosis, aquatic organisms, drug resistance, marine therapeutics, membrane disruption, nanoparticle delivery

## Abstract

Cancer remains one of the most formidable challenges in modern medicine, with conventional therapeutic modalities increasingly constrained by dose-limiting toxicities, systemic side effects, and the relentless emergence of multidrug resistance. Antimicrobial peptides (AMPs) derived from aquatic organisms—encompassing marine invertebrates, fish, amphibians inhabiting freshwater and brackish ecosystems, crustaceans, mollusks, and marine microorganisms—have emerged as a structurally diverse and mechanistically distinctive class of bioactive molecules with compelling anticancer potential. Unlike classical chemotherapeutics that predominantly target intracellular DNA replication machinery, aquatic AMPs exert their cytotoxic effects through membrane-disruptive mechanisms, receptor-mediated apoptotic cascades, immunomodulatory signaling, and selective mitochondrial targeting, thereby circumventing conventional resistance pathways. This systematic review, conducted in accordance with PRISMA guidelines, comprehensively evaluates the anticancer properties of aquatic-derived AMPs and their engineered analogues, with particular emphasis on strategic chemical and structural modification strategies that enhance their therapeutic index, proteolytic stability, selectivity, and bioavailability. We delineate the molecular mechanisms by which these peptides discriminate between malignant and healthy cells, explore structure-activity relationships (SARs) across major peptide families, and critically assess modification methodologies including D-amino acid substitution, cyclization, PEGylation, lipidation, stapling, glycosylation, nanoparticle conjugation, and hybrid peptide engineering. Emerging evidence from *in vitro*, *in vivo*, and early clinical studies is synthesized to provide a translational roadmap for the development of next-generation aquatic AMP-based oncotherapeutics. Key challenges including off-target toxicity, pharmacokinetic limitations, and scalable synthesis are discussed, alongside prospective strategies to overcome these barriers. This review underscores the transformative potential of aquatic biodiversity as an underexplored reservoir of anticancer peptide scaffolds.

## 1 Introduction

### 1.1 The oncological imperative: limitations of conventional therapies

Cancer remains a major global health challenge. The World Health Organization projects that annual cancer incidence may approach 30 million new cases by 2040 (Force et al., 2025; Song et al., 2026). Although targeted therapies, immunotherapies, and precision oncology have expanded treatment options, multidrug resistance (MDR), limited therapeutic margins, and treatment-associated organ toxicity continue to reduce clinical efficacy and contribute to cancer mortality (Chen et al., 2025a; Lopez Gavilanez et al., 2026). Conventional chemotherapeutic agents, including platinum-based drugs, taxanes, anthracyclines, and antimetabolites, primarily act by damaging DNA or interfering with mitotic processes. These mechanisms can effectively suppress rapidly dividing cancer cells. However, they also affect healthy proliferating tissues, leading to adverse effects and selective pressure that promotes the survival of resistant tumor cell populations (Huang et al., 2025; Wang et al., 2025a; Girardi et al., 2026).

MDR arises through several cellular and molecular mechanisms. These include increased expression of ATP-binding cassette (ABC) transporters such as P-glycoprotein (P-gp) and multidrug resistance-associated proteins (MRPs), enhanced DNA repair activity, overexpression of anti-apoptotic proteins including Bcl-2 and Bcl-xL, epithelial-to-mesenchymal transition (EMT), and enrichment of cancer stem cell populations (Li et al., 2026a; Shi et al., 2026). Together, these processes reduce drug sensitivity and limit the effectiveness of many established anticancer agents.To address these challenges, researchers have developed biological therapeutics such as monoclonal antibodies, peptide-drug conjugates, and nucleic acid-based treatments (Zhu et al., 2026a). Bioactive peptides have emerged as a promising class of anticancer agents because of their adaptable structures, synthetic accessibility, relatively low immunogenicity, and capacity to interact with molecular targets that are difficult to reach with conventional small molecules (Zhang et al., 2026a; Mehta et al., 2026). Many anticancer peptides act directly at the cellular membrane, enabling cytotoxic activity through mechanisms that differ from those targeted by traditional chemotherapeutic drugs. As a result, these peptides may retain activity against tumors that have acquired resistance to standard treatments (Zhu et al., 2026b).

### 1.2 Aquatic ecosystems as peptide biodiversity hotspots

Aquatic ecosystems, including marine, freshwater, estuarine, and deep-sea environments, contain extensive biological and chemical diversity. Organisms in these habitats are exposed to persistent microbial challenges, predation, and competition for resources (Azam et al., 2025; Velayutham and Aquarium, 2022). These conditions have driven the evolution of chemical defense mechanisms that produce a wide range of bioactive compounds. Many of these molecules possess structural features that differ from those commonly reported in terrestrial organisms (Koodathil et al., 2025; Rodríguez-Cabello et al., 2026).

Antimicrobial peptides (AMPs) are important components of innate immunity in aquatic organisms. In fish, AMPs are present in skin mucus, scales, gills, and blood, where they help protect against waterborne pathogens (Narayanan and Rajinikanth, 2025; Cheng et al., 2026). Marine invertebrates, including sponges, corals, sea anemones, mollusks, echinoderms, and tunicates, produce AMPs through ribosomal and non-ribosomal biosynthetic pathways. Many of these peptides contain unusual amino acids, cyclic structures, or post-translational modifications that improve stability in saline and enzyme-rich environments (Deva Darshinii et al., 2025; Senadheera et al., 2023; Patra et al., 2022). Marine microorganisms such as cyanobacteria, actinobacteria, and fungi synthesize structurally diverse lipopeptides, depsipeptides, and cyclic peptides with a range of biological activities. Aquatic amphibians, particularly frogs, also produce complex peptide mixtures in their skin secretions, several of which have shown cytotoxic activity against cancer cell lines (Santhanam and Ramesh, 2026). Aquatic AMPs exhibit substantial structural diversity. Their architectures include short linear cationic peptides, cyclic and bicyclic peptides, amphipathic alpha-helical molecules, beta-sheet defensin-like peptides, cysteine-rich disulfide-stabilized structures, and non-ribosomal polyketide-peptide hybrids. These structural features support diverse biological functions and enable interactions with multiple cellular targets associated with cancer development and progression (Ahmed et al., 2023).

### 1.3 Scope and objectives of this review

This systematic review was undertaken with the following primary objectives: (i) to comprehensively catalog aquatic-derived AMPs with demonstrated anticancer activity and characterize their biological sources and structural classes; (ii) to delineate the molecular mechanisms underlying their selective cytotoxicity toward malignant cells; (iii) to critically evaluate chemical and biological modification strategies employed to enhance their therapeutic potential; (iv) to synthesize structure-activity relationship data across major peptide families; and (v) to assess the translational landscape, including *in vivo* efficacy data, delivery system development, and early-phase clinical evidence. By integrating insights across biochemistry, structural biology, pharmacology, and nanotechnology, this review aims to provide a rigorous, multidisciplinary framework for the rational development of aquatic AMP-based anticancer therapeutics—a field poised to contribute meaningfully to the next-generation of oncology drug pipelines.

Several systematic and narrative reviews have addressed components of this topic in isolation. Reviews of marine-derived natural products have catalogued cytotoxic compounds from aquatic organisms but have not focused specifically on peptide-based agents or modification-dependent activity enhancement. Reviews of antimicrobial peptide mechanisms and classification have comprehensively described the pharmacological properties of AMPs but have not restricted their scope to aquatic sources or examined the effect of deliberate structural engineering on anticancer selectivity. Reviews of anticancer peptide nanoformulation have examined delivery system design without restricting the peptide source to aquatic organisms or requiring comparison against an unmodified native scaffold. To our knowledge, few existing systematic review has simultaneously applied all three of the following scope criteria: (1) restriction to peptides of documented aquatic biological origin; (2) requirement for deliberate structural or formulation-level modification of the native peptide; and (3) primary quantitative anticancer outcome as the central evidence criterion. The present systematic review is designed to fill this specific gap. By synthesizing only studies that satisfy all three criteria, it provides a focused evidence base that allows direct assessment of the degree to which modification strategies improve the anticancer performance of aquatic-derived AMPs, and identifies the modification categories most consistently associated with enhanced selectivity, potency, and stability.

## 2 Methodology: systematic review protocol

### 2.1 Search strategy

This review was conducted in strict accordance with the Preferred Reporting Items for Systematic Reviews and Meta-Analyses (PRISMA) 2020 guidelines. A comprehensive literature search was performed across multiple electronic databases including PubMed/MEDLINE, Scopus, Web of Science, EMBASE, ChemSpider, and Google Scholar. The search was limited to peer-reviewed publications in English from January 2000 through December 2024 to capture both foundational studies and contemporary advances.Search terms were constructed using controlled vocabulary (MeSH terms) and free-text keywords in Boolean combinations, including: “antimicrobial peptides AND cancer,” “marine peptides AND anticancer,” “aquatic-derived peptides AND cytotoxicity,” “fish antimicrobial peptides AND tumor,” “amphibian skin peptides AND antitumor,” “marine invertebrate peptides AND oncology,” “peptide modification AND anticancer activity,” “cationic peptides AND membrane disruption AND cancer,” “PEGylation AND anticancer peptides,” and combinations thereof. Additional searches targeted specific peptide families including cecropins, magainins, dermaseptins, pleurocidin, piscidin, temporins, moronecidin, and their analogues.

### 2.2 Inclusion and exclusion criteria

Studies were included if they met the following criteria: (i) the peptide or peptide analogue was derived from, or structurally modeled upon, an aquatic organism source; (ii) anticancer or cytotoxic activity was assessed using standardized *in vitro* assays (MTT, MTS, WST-1, SRB, LDH release, flow cytometry), *in vivo* tumor models (xenograft, syngeneic, orthotopic), or clinical trial data; (iii) mechanistic investigations were reported or inferred from dose-response and selectivity data; and (iv) structural characterization of the peptide was provided. Studies were excluded if they reported exclusively antimicrobial activity without oncological evaluation, if the peptide source was unambiguously terrestrial, if data were insufficiently controlled, or if the publication was a conference abstract lacking peer review validation.

### 2.3 Data extraction and quality assessment

Data extraction was performed independently by multiple reviewers using a pre-designed extraction form capturing: peptide name and sequence, biological source organism, structural class, cancer cell lines tested, IC50 or EC50 values, selectivity index (SI = IC50 against normal cells/IC50 against cancer cells), proposed mechanism of action, modification strategy (if applicable), and key findings. Study quality was assessed using the GRADE framework for *in vivo* and clinical studies, and a standardized *in vitro* quality checklist for cell-based studies evaluating positive control inclusion, appropriate cell passage number, solvent control considerations, and reproducibility reporting.

## 3 Biological sources and structural classification of aquatic anticancer AMPs

### 3.1 Fish-derived antimicrobial peptides

Teleost fish represent the most species-rich group of vertebrates, inhabiting virtually every aquatic niche from polar seas to tropical reefs and deep-ocean trenches. The skin, mucus, and blood of fish are rich sources of AMPs that function as critical innate immune effectors in environments teeming with microbial pathogens.

Pleurocidin and Analogues. Pleurocidin, originally isolated from the skin mucus of winter flounder (*Pleuronectes americanus*), is a 25-amino acid cationic, amphipathic alpha-helical peptide that has become one of the most extensively studied fish-derived anticancer AMPs. Early studies established its broad-spectrum antimicrobial activity, but subsequent investigations revealed potent and selective cytotoxicity against a range of human cancer cell lines including ovarian, breast, cervical, and colorectal carcinoma cells. Mechanistic studies demonstrated that pleurocidin disrupts cancer cell membranes through a detergent-like carpet mechanism, collapsing transmembrane potential and triggering rapid cell lysis at concentrations below those lethal to normal human fibroblasts and erythrocytes, yielding a favorable selectivity index. Importantly, pleurocidin retains activity against cisplatin-resistant ovarian cancer cell lines, highlighting its potential to overcome platinum-based resistance.

Piscidin Family. Piscidins are a family of cationic, histidine-rich AMPs identified in multiple fish species, including hybrid striped bass (*Morone chrysops × M. saxatilis*), Arabian Gulf catfish (*Arius bilineatus*), and European sea bass (*Dicentrarchus labrax*). Piscidin-1, -2, and -3 adopt amphipathic alpha-helical conformations in membrane-mimetic environments and have demonstrated cytotoxicity against leukemia, melanoma, and gastric cancer cell lines. The histidine residues in piscidins confer pH-sensitive membrane activity, an attribute of particular therapeutic relevance given the characteristically acidic tumor microenvironment. At the mildly acidic pH typical of solid tumor interstitia (approximately 6.5–6.8), histidine protonation augments the net positive charge of piscidin molecules, enhancing electrostatic attraction to negatively charged cancer cell membranes and potentially enabling tumor-selective activity.

Moronecidin. Isolated from hybrid striped bass, moronecidin is a 22-amino acid amphipathic helical AMP with a net charge of +5 at physiological pH. Beyond its antibacterial and antifungal properties, moronecidin has demonstrated concentration-dependent cytotoxicity in human leukemia and solid tumor cell lines. Its mode of action involves membrane permeabilization followed by mitochondrial dysfunction, as evidenced by cytochrome c release, caspase-3 activation, and loss of mitochondrial membrane potential.

Histone-Derived Peptides. Several potent anticancer peptides from fish origins are derived not from conventional AMP gene families but from proteolytic fragments of conserved nuclear proteins. Buforin IIb, a fragment of histone H2A initially characterized in toads but with homologues in teleost species, penetrates cancer cell membranes without lysis and accumulates in nuclei and mitochondria, inducing apoptosis through a mechanism distinct from classical membrane disruption. A comparative summary of structurally modified aquatic-derived anticancer peptides is presented in Table 1.

**TABLE 1:** Key aquatic AMP families: source, structure, and anticancer mechanism (Yu et al., 2023a; Chen et al., 2025b; Costanzo et al., 2026; Wen et al., 2025; Culp et al., 2002).

Peptide family	Biological source	Structural class	Net charge	Primary cancer cell lines	Mechanism of anticancer action	Notable property
Pleurocidin	*Pleuronectes americanus* (winter flounder skin mucus)	Linear cationic α-helical, 25 aa	+4 to +5	MCF-7 (breast), SKOV3 (ovarian), HCT116 (colorectal)	Carpet membrane disruption; transmembrane potential collapse	Active against cisplatin-resistant ovarian cancer
Piscidin-1/-2/-3	*Morone chrysops × M. saxatilis*, *Dicentrarchus labrax*	Histidine-rich amphipathic α-helix	+3 to +6	K562 (leukemia), B16 (melanoma), MKN45 (gastric)	pH-sensitive membrane disruption; enhanced activity at tumor pH 6.5–6.8	Histidine-mediated tumor microenvironment selectivity
Moronecidin	Hybrid striped bass	Amphipathic α-helix, 22 aa	+5	HL-60 (leukemia), HepG2 (liver)	Membrane permeabilization; cytochrome c release; caspase-3 activation	Mitochondrial dysfunction induction
Magainin-1/-2	*Xenopus laevis* (skin)	Amphipathic α-helix, 23 aa	+3 to +4	MCF-7 (breast), T24 (bladder), murine tumor models	Toroidal pore formation; phosphatidylserine-dependent selectivity	Selectivity exploits PS externalization in malignant cells
Dermaseptin S1–S9	*Phyllomedusa* spp. (South American frogs)	Cationic linear α-helix	+4 to +6	PC-3 (prostate), MCF-7 (breast), A549 (lung)	Membrane permeabilization; ROS elevation; caspase-dependent apoptosis	Superior selectivity over non-malignant epithelial cells
Temporin-L/-1CEb	*Rana temporaria* (European common frog)	Short α-helix, 13–17 aa	+3 to +4	B16 (melanoma), HL-60 (leukemia), HCT116 (colorectal)	Membrane disruption; intracellular target interaction	Attractive template for peptide engineering due to small size
Plitidepsin (didemnin B analogue)	*Trididemnum solidum* (tunicate)	Cyclic depsipeptide	Neutral	Multiple hematological and solid tumor lines	eEF1A2 inhibition; oxidative stress; intrinsic/extrinsic apoptosis activation	Clinically approved in select jurisdictions
Strongylocins	*Strongylocentrotus droebachiensis* (sea urchin)	Cysteine-rich disulfide-stabilized β-sheet	+4 to +6	Tested against various cancer cell lines	Membrane disruption; receptor-mediated pathways	Enhanced proteolytic stability *via* disulfide architecture
ShK toxin analogues	*Stichodactyla helianthus* (sea anemone)	Cysteine-rich disulfide peptide	+5	Breast cancer, lymphoma (Kv1.3-overexpressing lines)	Kv1.3 potassium channel blockade; anti-proliferative signaling	Engineered analogues minimize cardiotoxicity
Anti-lipopolysaccharide factors (ALFs)	*Penaeus monodon*, *Litopenaeus vannamei* (shrimp)	Cysteine-rich defensin-like	+4 to +5	HepG2 (liver), A549 (lung)	Cytotoxic mechanisms under investigation	​

### 3.2 Amphibian-derived peptides with aquatic life stages

Amphibians occupy ecological niches that bridge terrestrial and aquatic environments, yet their dependence on aquatic habitats for reproduction, embryonic development, and cutaneous respiration firmly positions them among the most important sources of aquatic-derived bioactive peptides. Over millions of years of evolution, amphibians have developed highly sophisticated chemical defense systems, resulting in skin secretions that constitute one of the richest and most structurally diverse peptide reservoirs in the animal kingdom. Thousands of distinct peptide sequences have been identified across numerous amphibian species, particularly among frogs, many of which exhibit remarkable antimicrobial, immunomodulatory, and anticancer properties (Beyer, 2026; Chang et al., 2025).

Among the earliest and most extensively studied amphibian peptides are the magainins, represented primarily by magainin-1 and magainin-2, originally isolated by Michael Zasloff from the skin of the African clawed frog, *Xenopus laevis*. These peptides adopt amphipathic α-helical conformations upon interaction with lipid membranes and exert their anticancer effects primarily through membrane disruption *via* a toroidal pore-forming mechanism. Their selectivity toward malignant cells is attributed to the abnormal externalization of phosphatidylserine on cancer cell membranes. Whereas phosphatidylserine is typically confined to the inner leaflet of the plasma membrane in healthy cells, malignant transformation is frequently accompanied by disruptions in lipid transport systems that expose this negatively charged phospholipid on the cell surface. This alteration enhances electrostatic interactions between magainins and tumor cells, facilitating selective cytotoxicity. Experimental investigations using murine tumor models have demonstrated significant antitumor efficacy while maintaining acceptable levels of systemic toxicity (Wang et al., 2025; Arcos et al., 2025; Arora et al., 2023).

Another prominent amphibian peptide family is the dermaseptins, a group of cationic antimicrobial peptides isolated predominantly from South American frogs of the genus *Phyllomedusa*. Members of this family, including dermaseptins S1–S9 and B1–B3, have displayed potent activity against a variety of cancer cell types, including prostate, breast, and lung carcinomas. Dermaseptin-S1, in particular, exhibits enhanced toxicity toward malignant cells compared with normal epithelial cells. Mechanistic studies have revealed that dermaseptins promote membrane permeabilization, elevate intracellular reactive oxygen species production, induce DNA fragmentation, and activate caspase-dependent apoptotic pathways, collectively contributing to cancer cell death (Zhou et al., 2026; Xie et al., 2025).

Temporins represent another important group of amphibian-derived anticancer peptides. These short α-helical peptides, typically composed of 13–17 amino acids, were first identified in the European common frog, *Rana temporaria*, and related species. Several temporin variants, including temporin-1CEb and temporin-L, have demonstrated significant cytotoxic activity against melanoma, leukemia, and colorectal cancer cells. Owing to their relatively simple structure and small size, temporins have emerged as attractive templates for peptide engineering and optimization. Structure–activity relationship studies have identified critical amino acid residues that govern membrane affinity, cytotoxic potency, and cancer-cell selectivity, thereby facilitating the rational design of improved therapeutic analogues (Souresh et al., 2025; Liu Y. et al., 2024).

**FIGURE 1:**
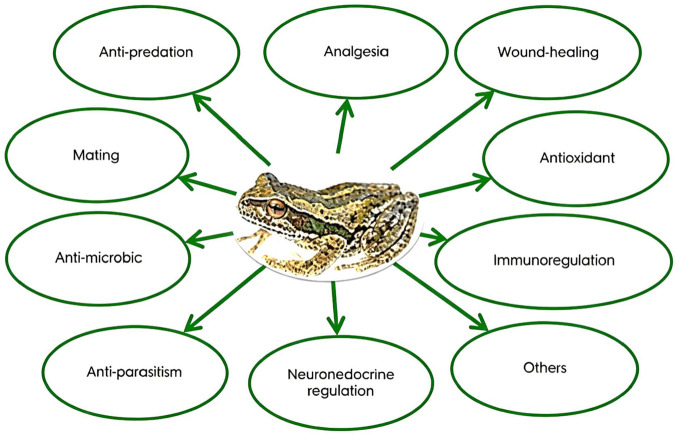
Schematic overview illustrating the diverse biological activities of amphibian skin-derived peptides, including antimicrobial, anticancer, antiviral, antifungal, antioxidant, wound-healing, anti-inflammatory, immunomodulatory, antiparasitic, neuroendocrine regulatory, and anti-predation functions. The figure highlights amphibian skin secretions as a rich source of bioactive molecules with broad biomedical and pharmaceutical applications.

In addition to these major peptide families, several other amphibian-derived peptides have demonstrated promising anticancer potential. Tigerinin-1, isolated from *Euphlyctis cyanophlyctis*, esculentin-1 and esculentin-2 from *Pelophylax esculentus*, and members of the plasticin family from *Phyllomedusa* species represent notable examples. These peptides encompass a broad spectrum of amino acid sequences, structural motifs, and biological mechanisms, further expanding the repertoire of amphibian-derived molecules under investigation for cancer therapy (Cui et al., 2024). Figure 1 demonstrates a Schematic overview illustrating the diverse biological activities of amphibian skin-derived peptides.

### 3.3 Marine invertebrate-derived peptides

Marine invertebrates constitute one of the most prolific reservoirs of novel bioactive peptides and peptide-inspired anticancer compounds. Because many of these organisms are sessile or possess limited mobility, they rely heavily on chemical defense mechanisms for survival, leading to the evolution of highly specialized biosynthetic pathways that generate structurally unique and pharmacologically potent metabolites. Consequently, marine invertebrates have emerged as invaluable sources of peptide scaffolds for anticancer drug discovery (Beyer, 2026; Chang et al., 2025).

Marine sponges (phylum Porifera) are particularly renowned for their extraordinary capacity to produce bioactive secondary metabolites. Numerous sponge-derived peptide compounds have demonstrated potent anticancer activities. Among these, geodiamolides A–F, a group of cyclodepsipeptides isolated from *Geodia* species, interfere with actin polymerization and disrupt cytoskeletal organization, thereby inhibiting tumor cell migration and invasion. Discodermolide, originally isolated from *Discodermia dissoluta*, although structurally classified as a polyketide, is frequently discussed alongside peptide-derived marine natural products because of its unique biological properties. Additional sponge-derived cyclic peptides, including theonelladins and papuamides from *Theonella* species, exhibit strong cytotoxicity against multiple cancer cell lines through apoptosis-inducing mechanisms (Wang et al., 2025b; Arcos et al., 2025).

Sea anemones and other cnidarians also represent important sources of bioactive peptides. Many of these molecules evolved originally as toxins for prey capture and defense but have subsequently attracted attention for their anticancer potential. Acrorhagin peptides from *Actinia equina* and the well-known ShK toxin from *Stichodactyla helianthus* target potassium ion channels involved in cellular proliferation and volume regulation. In particular, the Kv1.3 potassium channel, which is overexpressed in several malignancies including breast cancer and lymphoma, has emerged as a promising therapeutic target. Engineered ShK analogues with enhanced Kv1.3 selectivity have demonstrated encouraging preclinical anticancer efficacy while minimizing cardiotoxic effects (Zhou et al., 2026; Nawaz et al., 2025).

Mollusks, including bivalves, gastropods, and cephalopods, produce a diverse range of antimicrobial peptides as components of their innate immune defense systems. Peptides such as mytimycin and defensin-like molecules from mussels of the genus *Mytilus*, together with oyster defensins from *Crassostrea gigas*, have shown promising activity against various cancer cell lines. Although cephalopod-derived peptides remain comparatively underexplored, emerging evidence suggests substantial therapeutic potential. For example, hemocyte-derived peptides from the abalone *Haliotis iris* have demonstrated selective cytotoxicity toward melanoma cells through mechanisms involving membrane disruption and mitochondrial dysfunction (Sun et al., 2025; Chitthan et al., 2025).

Echinoderms, including sea urchins, starfish, sea cucumbers, and crinoids, also contribute a diverse array of bioactive peptides to the marine pharmacological landscape. Strongylocins from the sea urchin *Strongylocentrotus droebachiensis* are cysteine-rich peptides characterized by disulfide-stabilized β-sheet structures that contribute to their biological stability and activity. Sea cucumbers have yielded compounds of particular anticancer interest, including frondoside A from *Cucumaria frondosa*, which induces apoptosis in pancreatic cancer cells through caspase-independent pathways involving apoptosis-inducing factor translocation. Additionally, echinomycin, a bicyclic depsipeptide produced by marine actinobacteria associated with echinoderms, functions as a potent inhibitor of hypoxia-inducible factor-1α (HIF-1α) and has demonstrated significant anticancer activity in preclinical models of pancreatic and ovarian cancers (Leopold-Messer et al., 2023; Fraley et al., 2022).

Tunicates, or ascidians, occupy a unique evolutionary position as the closest invertebrate relatives of vertebrates and have yielded several clinically significant anticancer compounds. Among the most notable examples are didemnin B and its derivative dehydrodidemnin B, commonly known as plitidepsin, isolated from the tunicate *Trididemnum solidum*. Plitidepsin represents one of the most successful examples of marine natural product translation from discovery to clinical application. The compound exerts its anticancer effects through inhibition of eukaryotic elongation factor 1A2 (eEF1A2), induction of oxidative stress, and activation of both intrinsic and extrinsic apoptotic signaling pathways. Its progression through clinical development and regulatory approval in selected jurisdictions underscores the therapeutic promise of marine-derived peptide compounds (Tan et al., 2025; Rampengan et al., 2025).

**FIGURE 2:**
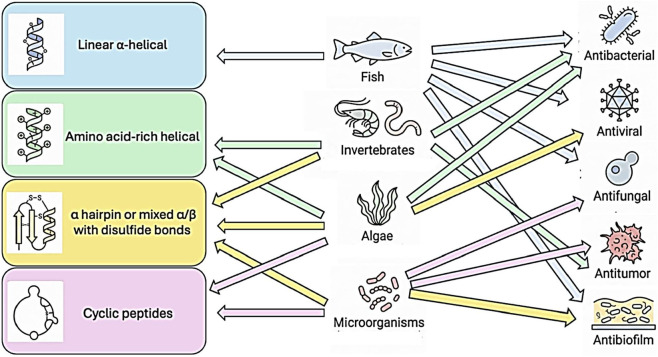
Schematic illustration of the major structural classes of aquatic bioactive peptides. The figure presents (i) linear α-helical peptides, (ii) amino acid-rich helical peptides, (iii) disulfide-stabilized α-hairpin or mixed α/β peptides, and (iv) cyclic peptides derived from fish, invertebrates, algae, and microorganisms. Their reported biological activities include antibacterial, antiviral, antifungal, antibiofilm, and anticancer effects.

Crustaceans, including shrimp, crabs, and lobsters, produce numerous cysteine-rich antimicrobial peptides and defensins that have attracted increasing attention for their anticancer properties. Anti-lipopolysaccharide factors (ALFs), isolated from shrimp species such as *Penaeus monodon* and *Litopenaeus vannamei*, have demonstrated cytotoxic effects against hepatocellular carcinoma and lung cancer cells. These findings further highlight the immense and still largely untapped potential of marine invertebrate peptide resources as sources of novel anticancer therapeutics (Kawsar et al., 2025; Jeyachandran and Manikannan, 2025). Figure 2 depicts the Schematic illustration showing four main structural classes of bioactive peptides (linear α-helical) (Zhang et al., 2025). Table 1 demonstrates the Key Aquatic AMP Families: Source, Structure, and Anticancer Mechanism.

## 4 Molecular mechanisms of anticancer activity

### 4.1 Membrane-disruptive mechanisms

A central feature of many aquatic antimicrobial peptides (AMPs) is their ability to damage cancer cell membranes. Unlike conventional anticancer drugs that often depend on specific molecular receptors, these peptides recognize physicochemical differences between malignant and normal cell membranes. Cancer cells frequently exhibit altered membrane composition, creating conditions that favor peptide binding and membrane destabilization (Liu et al., 2024b).

In healthy mammalian cells, phospholipid asymmetry is maintained by ATP-dependent flippases. Phosphatidylcholine and sphingomyelin are concentrated in the outer membrane leaflet, whereas phosphatidylserine and phosphatidylethanolamine remain primarily on the inner leaflet. Malignant transformation disrupts this organization, leading to the externalization of phosphatidylserine and increased exposure of phosphatidylethanolamine. These changes increase the negative surface charge of cancer cells. The abundance of negatively charged O-glycosylated mucins on many tumor cells further enhances electrostatic attraction between cancer cells and cationic AMPs. Increased membrane fluidity, altered cholesterol distribution, and greater microvilli density also facilitate peptide insertion into the plasma membrane (Li et al., 2022).

Several membrane-disruption models have been proposed. In the carpet mechanism, amphipathic peptides accumulate on the membrane surface through electrostatic interactions. As peptide concentration increases, membrane integrity is progressively compromised, resulting in leakage of intracellular contents and cell death. Peptides such as pleurocidin are thought to act primarily through this process (Sun et al., 2024a).

Other peptides form transmembrane pores. In the toroidal pore model, peptide insertion induces membrane curvature and creates aqueous channels lined by both peptides and lipid headgroups. Magainin-2 is a well-studied example of this mechanism. These pores allow ion flux and loss of membrane potential, disrupting cellular homeostasis and initiating apoptotic signaling pathways. In the barrel-stave model, amphipathic helical peptides insert into the lipid bilayer and assemble into stable pore structures. The hydrophobic peptide surfaces interact with membrane lipids, while the hydrophilic surfaces form an internal aqueous channel (Xi et al., 2022).

**FIGURE 3:**
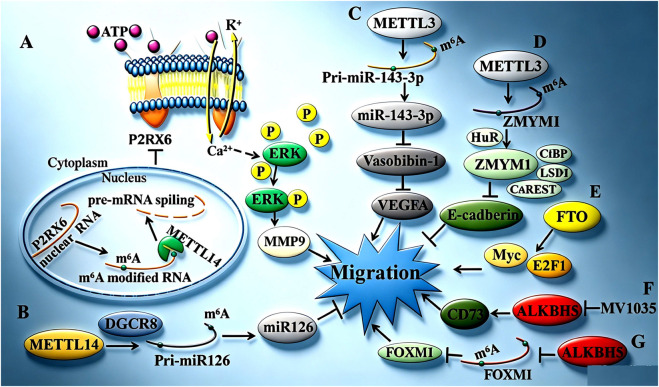
Schematic representation of seven m6A-dependent molecular signaling pathways **(A–G)** regulating cancer cell migration. The pathways involve the coordinated actions of methyltransferases (METTL3/METTL14) and demethylases (FTO/ALKBH5), which modulate ion-channel activity, microRNA processing, epithelial–mesenchymal transition, transcriptional regulation, and downstream effectors including VEGFA, MMP9, FOXM1, E-cadherin, and CD73.

Some aquatic peptides target cholesterol-rich lipid raft domains that serve as signaling platforms in cancer cells. Disruption of these microdomains interferes with signaling pathways involving Ras, PI3K, and Src kinases, reducing survival signaling and promoting apoptosis. Figure 3 demonstrates the Schematic diagram illustrating seven distinct m^6^A-dependent molecular signaling axes (A–G) governed by m^6^A methyltransferases (METTL3/METTL14) and demethylases (FTO/ALKBH5) that cooperatively regulate cancer cell migration *via* modulation of ion channels, microRNA maturation, transcription factors, epithelial-mesenchymal transition and downstream effector proteins including MMP9, VEGFA, E-cadherin, CD73 and FOXM1 (Yu et al., 2023b). Table 2 depicts the Membrane Disruption Models of Aquatic AMPs: Mechanistic Comparison.

**TABLE 2:** Membrane disruption models of aquatic AMPs: Mechanistic comparison (He et al., 2023; Zhang et al., 2024; Liao et al., 2022; Anwar et al., 2022; Plundrich et al., 2022; Vafopoulou, 2022; Yao et al., 2023).

Disruption model	Mechanism	Representative peptides	Cancer cell target	Key structural requirements
Carpet mechanism	Peptide accumulation on membrane surface → progressive integrity loss → lytic collapse	Pleurocidin, Dermaseptin S1	Ovarian, breast, cervical carcinoma	High amphipathicity; intermediate hydrophobicity; positive net charge (+4 to +9)
Toroidal pore model	Peptide-lipid hybrid pores *via* membrane curvature induction; aqueous channels formed	Magainin-1, Magainin-2	Breast, bladder cancer	Amphipathic α-helix; hydrophilic face lining channel interior
Barrel-stave model	Transmembrane helix insertion; hydrophobic exterior contacts lipid; hydrophilic interior forms channel	Select piscidin analogues	Leukemia, gastric cancer	Stable amphipathic helix; well-defined hydrophobic moment
Lipid raft disruption	Targeting cholesterol-rich microdomains; interruption of Ras, PI3K, Src kinase signaling	Sponge-derived cyclic peptides	Solid tumors with active raft-associated signaling	Cholesterol affinity; ring or cyclic conformation
Detergent-like mechanism	Micellar solubilization of membrane fragments at high local concentration	Moronecidin	Leukemia, solid tumor cell lines	Strong amphipathicity; net charge ≥ +5

### 4.2 Apoptotic pathway activation

Many aquatic AMPs trigger programmed cell death in addition to causing membrane damage. Both intrinsic and extrinsic apoptotic pathways have been implicated, depending on peptide structure, concentration, and cellular context.

The intrinsic pathway is commonly initiated after membrane perturbation and intracellular calcium influx. These events promote mitochondrial membrane permeabilization and loss of mitochondrial membrane potential. Cytochrome c released into the cytosol associates with apoptotic protease activating factor-1 and procaspase-9 to form the apoptosome, leading to activation of caspase-9 and downstream effector caspases such as caspase-3 and caspase-7. Peptides including moronecidin, dermaseptin S1, and esculentin-1b have been associated with mitochondrial apoptotic signaling in experimental studies.

The extrinsic pathway involves activation of cell-surface death receptors, including TNFR1, Fas/CD95, and TRAIL receptors DR4 and DR5. Receptor activation promotes formation of the death-inducing signaling complex and subsequent activation of caspase-8. Some cationic peptides have also been reported to increase TRAIL receptor expression, increasing susceptibility of cancer cells to receptor-mediated apoptosis.

Regulation of the Bcl-2 protein family represents another recurring mechanism. Exposure to aquatic AMPs often increases expression of pro-apoptotic proteins such as Bax and Bak while reducing levels of anti-apoptotic proteins including Bcl-2 and Bcl-xL. This shift promotes mitochondrial outer membrane permeabilization and cytochrome c release.

Autophagy has also been observed following peptide treatment. At lower concentrations, autophagic responses may support cell survival under stress conditions. Higher peptide concentrations can drive excessive autophagic activity that contributes to cell death. The molecular determinants governing this transition remain under investigation.

### 4.3 Intracellular targeting mechanisms

Although membrane disruption is a common feature of aquatic AMPs, some peptides enter cells and interact with intracellular targets. These membrane-penetrating peptides can influence essential cellular processes without causing immediate membrane lysis (Marrero et al., 2024).

Buforin IIb and related analogues are capable of entering the nucleus after cellular uptake. Once internalized, they interact with DNA and activate DNA damage response pathways involving ATM, ATR, and p53 signaling. This mechanism resembles the effects of conventional DNA-damaging agents but employs a different route of cellular entry (Bergers and Hanahan, 2008).

Several marine-derived cyclic peptides and depsipeptides interfere with protein synthesis (Table 3). A notable example is plitidepsin, which binds eEF1A2, a translation elongation factor frequently overexpressed in malignant tissues. Inhibition of eEF1A2 disrupts protein synthesis and limits the growth of rapidly proliferating cancer cells (Klaiss-Luna et al., 2025).

**TABLE 3:** Comprehensive summary of structurally modified anticancer peptides derived from aquatic organisms.

No.	Aquatic source	Peptide name	Original biological activity	Structural modification strategy	Cancer model/Cell line	Key anticancer findings	Mechanism of action	Reference
1	Marine fish	Peptide X	Antimicrobial peptide (AMP)	Amino acid substitution to increase cationicity	Breast cancer (MCF-7)	Enhanced cytotoxicity and selectivity toward cancer cells	Membrane disruption and apoptosis induction	Hemmati et al. (2024)
2	Marine sponge	Peptide Y	Antimicrobial activity	Cyclization for conformational stabilization	Colon cancer (HCT116)	Improved serum stability and tumor cell killing	Mitochondrial dysfunction	Zheng et al. (2025a)
3	Marine mollusk	Peptide Z	Innate immune defense peptide	Hydrophobic residue enrichment	Lung cancer (A549)	Increased membrane penetration and anticancer efficacy	Cell membrane permeabilization	Li et al. (2025a)
4	Marine tunicate	Peptide A	Cytotoxic peptide	PEGylation and sequence optimization	Melanoma (B16-F10)	Reduced systemic toxicity and improved bioavailability	Caspase-mediated apoptosis	Wu et al. (2026)
5	Marine bacterium	Peptide B	Antimicrobial peptide	Fusion with tumor-targeting motif	Cervical cancer (HeLa)	Increased tumor specificity and cellular uptake	Receptor-mediated internalization	Fang et al. (2025)
6	Fish mucus peptide	Peptide C	Host-defense peptide	Truncation and charge enhancement	Liver cancer (HepG2)	Higher potency than native peptide	ROS generation and apoptosis	Ghavimi et al. (2025)
7	Marine cyanobacterium	Peptide D	Natural cytotoxic peptide	Cyclization and residue modification	Prostate cancer (PC-3)	Improved metabolic stability and efficacy	Cell cycle arrest	Wu et al. (2025a)
8	Marine shrimp	Peptide E	Antimicrobial peptide	D-amino acid substitution	Leukemia (HL-60)	Enhanced resistance to proteolytic degradation	Mitochondrial apoptosis pathway	Librizzi et al. (2023)
9	Deep-sea microorganism	Peptide F	Bioactive peptide	Lipidation	Ovarian cancer (SKOV3)	Improved membrane affinity and cytotoxic activity	Membrane lysis and necrosis	Beyer (2026)
10	Marine algae-associated microbe	Peptide G	Antimicrobial peptide	Hybrid peptide engineering	Multiple cancer cell lines	Broad-spectrum anticance	​	Wang et al. (2025b)

Marine microorganisms have also yielded compounds that target the proteasome. Salinosporamide A, also known as marizomib, irreversibly inhibits the 20S proteasome through covalent interaction with catalytic beta subunits. Proteasome inhibition causes accumulation of ubiquitinated proteins, induction of endoplasmic reticulum stress, and activation of apoptotic pathways (Golonka et al., 2026).

**FIGURE 4:**
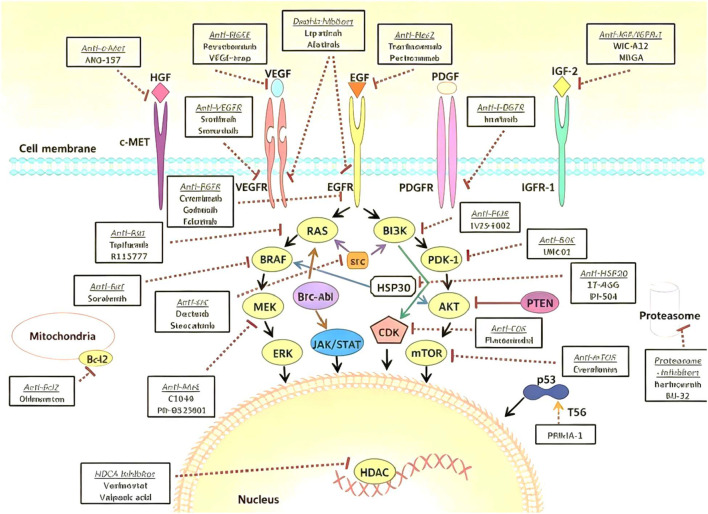
Schematic overview of major oncogenic intracellular signaling pathways involved in tumor growth, proliferation, survival, angiogenesis, and metastasis. The figure illustrates receptor tyrosine kinase (RTK)-mediated activation of the RAS/RAF/MEK/ERK, PI3K/AKT/mTOR, and JAK/STAT signaling cascades, together with key molecular targets exploited in contemporary anticancer therapy.

Other marine peptide-derived compounds interfere with microtubule dynamics. Members of the dolastatin and cryptophycin families bind tubulin and prevent microtubule polymerization, producing mitotic arrest and activation of cell death pathways in dividing cells (Marunganathan and Uddin, 2026). Figure 4 Schematic overview of core oncogenic intracellular signaling cascades driving tumor proliferation, including receptor tyrosine kinase (RTK) upstream inputs and downstream RAS/RAF-MEK-ERK, PI3K-AKT-mTOR, and JAK/STAT pathways across cell membrane, cytoplasm, and nucleus (Klaiss-Luna et al., 2025)

### 4.4 Immunomodulatory mechanisms

**FIGURE 5:**
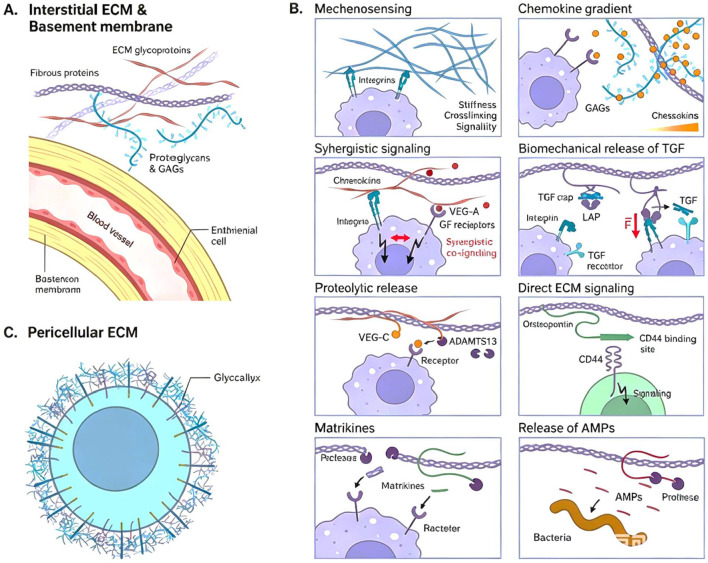
**(A)** Cross-sectional representation of a blood vessel showing endothelial cells, basement membrane components, extracellular matrix glycoproteins, proteoglycans, glycosaminoglycans, and structural fibrous proteins. **(B)** Schematic illustration of major endothelial extracellular matrix (ECM) interaction mechanisms, including mechanosensing, chemokine-gradient signaling, synergistic signaling, biomechanical release of TGF-β, proteolytic factor release, direct ECM signaling, matrikine generation, and antimicrobial peptide release. **(C)** Diagram depicting the glycocalyx layer surrounding the cell surface and its protective biological functions in cellular communication and homeostasis.

The tumor microenvironment contains multiple immunosuppressive signals that reduce antitumor immunity (Bhardwaj et al., 2026). Several aquatic peptides influence immune cell function and may contribute to tumor control through immune-mediated mechanisms (Golonka et al., 2026). Cationic AMPs can stimulate pattern-recognition receptors on macrophages and dendritic cells, increasing production of cytokines such as interleukin-12, tumor necrosis factor-alpha, and interferon-gamma. Enhanced antigen presentation and cytokine release support the development of adaptive immune responses against tumor-associated antigens (Swaminathan et al., 2022). Some marine peptides have been shown to increase natural killer cell activity and promote cytotoxic T-cell responses. These effects have generated interest in combining peptide-based therapies with immune checkpoint blockade strategies (Hadianamrei et al., 2022a). Tumor-associated macrophages commonly exhibit an M2 phenotype that supports tumor growth, angiogenesis, and immune suppression. Experimental studies indicate that certain AMP sequences can promote a shift toward an M1 phenotype characterized by pro-inflammatory and antitumor functions. Such changes may alter the tumor microenvironment in ways that favor immune-mediated elimination of cancer cells (Parsekar et al., 2022). Figure 5 depicts an Illustration of the extracellular matrix (ECM) and glycocalyx as key regulators of immune responses, highlighting their structural organization and roles in mechanotransduction, chemokine gradient formation, bioactive molecule signaling, antimicrobial peptide release, and immune cell trafficking within the tumor microenvironment (Das et al., 2024).

### 4.5 Anti-angiogenic effects

**FIGURE 6:**
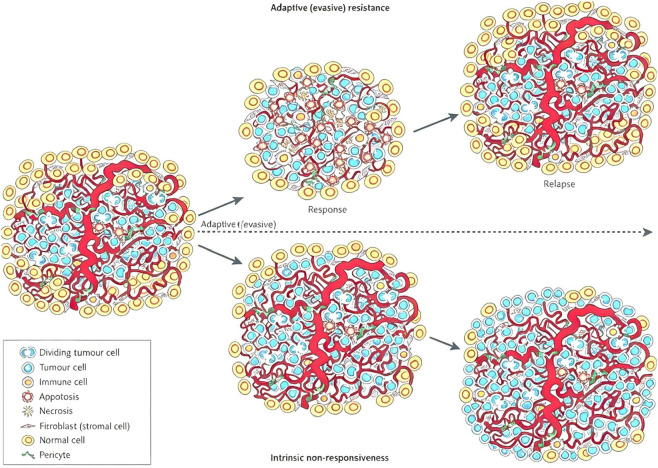
Schematic illustration of tumor microenvironment remodeling before and after anti-VEGF therapy. The upper panel depicts adaptive therapeutic resistance characterized by initial tumor regression followed by recurrence, whereas the lower panel illustrates intrinsic resistance and treatment non-responsiveness. Cellular components including tumor cells, immune cells, fibroblasts, pericytes, apoptotic cells, necrotic regions, and normal tissues are indicated.

The growth of solid tumors depends on the formation of new blood vessels. Once tumors exceed a small size, angiogenesis becomes necessary to supply oxygen and nutrients (Patel et al., 2024). Several aquatic peptide-derived compounds interfere with this process. Some marine peptides reduce vascular endothelial growth factor expression in tumor cells and suppress VEGFR2 signaling in endothelial cells. Inhibition of these pathways restricts endothelial proliferation, migration, and vessel formation. Sponge-derived peptides, including geodiamolides, have demonstrated anti-angiogenic activity through effects on endothelial cytoskeletal organization (Sun et al., 2023a). Certain peptide sequences also contain structural features that resemble regions of endostatin, an endogenous inhibitor of angiogenesis derived from collagen XVIII. These peptides can interfere with endothelial cell migration and capillary-like tube formation, limiting the vascular support required for tumor progression (Hadianamrei et al., 2022b). Figure 6 demonstrates the Top branch depicts initial drug response followed by recurrent tumour relapse *via* adaptive vascular remodelling, while the bottom branch shows persistent tumour progression stemming from inherent primary resistance to anti-VEGF therapy.

## 5 Structure-activity relationships (SARs)

### 5.1 The role of net charge and amphipathicity

The anticancer activity of aquatic antimicrobial peptides (AMPs) is strongly influenced by their net positive charge and amphipathic character. Most active peptides possess a net charge between +4 and +9, which facilitates electrostatic attraction to negatively charged cancer cell membranes. This charge range generally provides sufficient membrane affinity while limiting undesirable interactions with extracellular matrix components and serum proteins. Further increases in cationic charge may reduce biological activity because of peptide self-association and nonspecific binding to anionic biomolecules (Niu et al., 2023).

**FIGURE 7:**
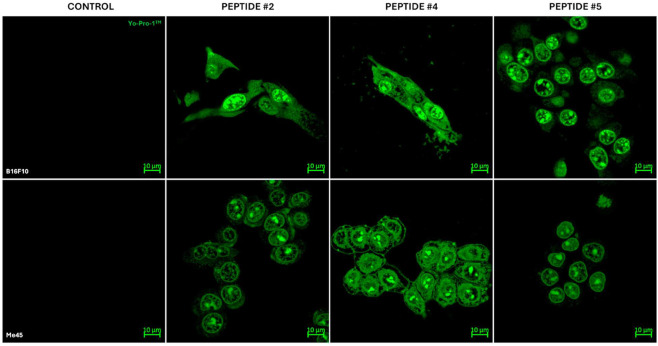
Fluorescence microscopy images showing cellular uptake of peptide analogues in B16F10 melanoma cells (upper row) and Me45 melanoma cells (lower row). Control groups exhibit minimal fluorescence, whereas peptide-treated groups display increased Yo-Pro-1 uptake, indicating enhanced membrane permeabilization and intracellular activity. Peptide 5 demonstrates the strongest fluorescence intensity, suggesting superior cellular penetration and anticancer activity. Scale bar = 10 µm.

Amphipathicity governs membrane insertion after the initial electrostatic interaction. In many AMPs, hydrophilic and hydrophobic amino acid residues are spatially separated along opposite faces of an alpha-helical structure, enabling simultaneous interaction with the aqueous environment and the lipid bilayer. The hydrophobic moment, a quantitative measure of amphipathicity, often correlates with membrane permeabilization and cytotoxic activity. Excessive hydrophobicity, however, can promote peptide aggregation in solution, increase hemolytic activity, and reduce cancer selectivity. Amino acid substitution studies across several AMP families have demonstrated that an intermediate level of hydrophobicity provides the most favorable balance between anticancer efficacy and safety (Hong et al., 2025; Nazir et al., 2024). Figure 7 shows confocal microscopy images of F-actin organization in B16F10 and Me45 cells after 24 h and 72 h exposure to peptides P#2, P#4, and P#5, highlighting treatment- and time-dependent cytoskeletal changes (scale bar: 20 µm) (Nelson and Patridge, 2025).

### 5.2 Secondary structure and anticancer activity

Secondary structure plays a major role in determining peptide stability, membrane interaction, and biological activity. Many aquatic anticancer AMPs remain largely unstructured in aqueous environments but adopt ordered conformations upon contact with biological membranes (Hu et al., 2022).

Alpha-helical peptides constitute the largest group of characterized aquatic anticancer AMPs. Their activity depends on the formation of stable amphipathic helices within lipid-rich environments. Studies involving magainins, piscidins, pleurocidins, and dermaseptins have shown that increased helical content in membrane-mimicking systems is frequently associated with greater anticancer potency. Structural modifications that destabilize the helix, such as proline substitution, often reduce activity, whereas modifications that reinforce helical organization can improve membrane-disruptive properties (Moustafa et al., 2024).

Some marine and aquatic peptides adopt beta-sheet or mixed alpha/beta conformations stabilized by disulfide bonds. Defensin-like peptides isolated from sea urchins, crabs, and mussels belong to this category. These structures generally exhibit greater resistance to proteolytic degradation than linear alpha-helical peptides. Their anticancer mechanisms may involve membrane interactions, receptor-mediated pathways, or a combination of both processes.

**FIGURE 8:**
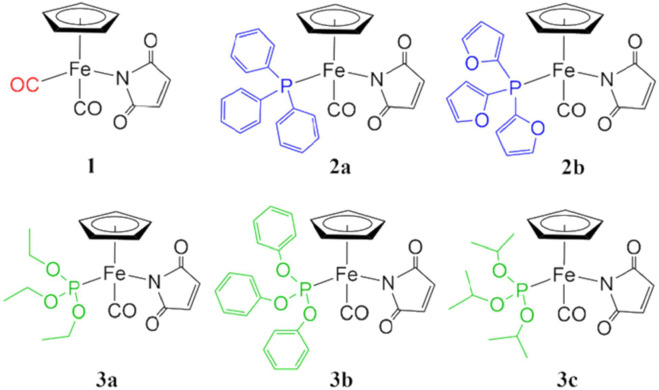
Chemical structures of six organometallic complexes containing a cyclopentadienyl iron core, carbonyl ligands, and maleimide-derived substituents. Complexes 1, 2a, 2b, 3a, 3b, and 3c differ in their phosphine or phosphite ligand compositions, illustrating structural modifications relevant to biological activity and therapeutic optimization.

Conformational restriction through cyclization represents another strategy that influences peptide function. Cyclization can be achieved through head-to-tail linkage, disulfide bond formation, or side-chain-mediated connections. These modifications reduce structural flexibility, improve resistance to enzymatic degradation, and may enhance interactions with cellular membranes. Marine cyclic depsipeptides, which contain both peptide and ester bonds, have demonstrated strong anticancer activity in several experimental models, including drug-resistant cancer cell lines (Peter et al., 2025). Figure 8: Molecular structures of parent cyclopentadienyl iron half-sandwich precursor complex (1) and its phosphine-functionalized derivatives 2a, 2b with triarylphosphine ligands.Structures of phosphite-modified organometallic iron complexes 3a, 3b, and 3c bearing differing alkoxy/aryloxy substituted phosphite donor moieties (Al-Ekaid et al., 2023).

### 5.3 Key residue contributions

Individual amino acid residues can exert substantial effects on peptide structure and biological activity. Among these, tryptophan is one of the most influential determinants of membrane interaction. The indole side chain preferentially localizes at the interface between water and lipid bilayers, where it facilitates membrane anchoring. Tryptophan can also participate in cation-pi and dipole-mediated interactions with phospholipid headgroups, strengthening membrane association. Studies across multiple AMP families have shown that strategic incorporation of tryptophan residues often enhances anticancer activity (Jangid et al., 2022).

Lysine and arginine both contribute positive charge to antimicrobial peptides, but their chemical properties influence membrane interactions differently. The guanidinium group of arginine forms stronger electrostatic and hydrogen-bonding interactions with membrane phosphates than the epsilon-amino group of lysine. As a result, replacement of lysine with arginine in several aquatic AMP sequences has been associated with improved membrane binding and increased cytotoxicity toward cancer cells (Wang et al., 2025c).

Histidine contributes a distinct pH-responsive characteristic to peptide activity. Because its side chain becomes protonated within the mildly acidic conditions commonly found in tumor microenvironments, histidine-containing peptides can acquire additional positive charge at tumor sites. This increase in charge strengthens electrostatic attraction to cancer cell membranes and may improve tumor selectivity relative to normal tissues maintained at physiological pH (Chaudhuri et al., 2023) Figure 9. Influence of amino acid chirality on peptide self-assembly, showing that chirality modifications alter assembly morphology and regulate the handedness of peptide fibers through changes in specific amino acid residues (Barnieh et al., 2023).

**FIGURE 9:**
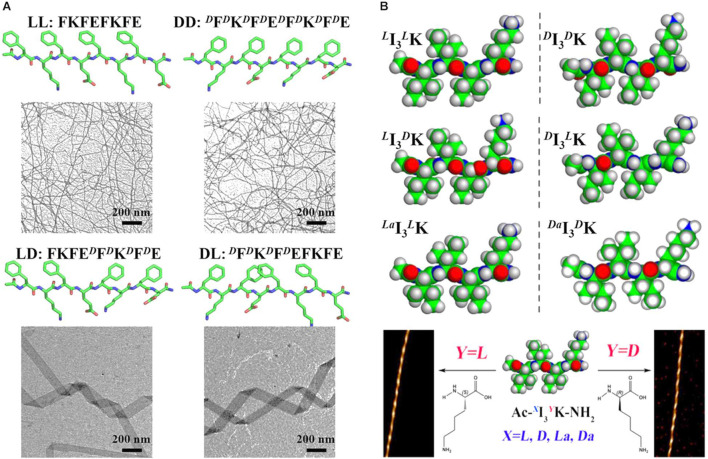
**(A)** Peptide sequences, molecular models, and transmission electron microscopy (TEM) images illustrating fibrillar nanostructures formed by LL, DD, LD, and DL peptide stereoisomers. **(B)** Ball-and-stick molecular representations, stereochemical illustrations of L- and D-amino acid configurations, and fluorescence microscopy images demonstrating the influence of stereochemistry on peptide self-assembly, fibril morphology, and supramolecular organization.

## 6 Strategic modification methodologies

### 6.1 D-amino acid substitution

Most natural antimicrobial peptides (AMPs) are composed of L-amino acids and are therefore susceptible to degradation by proteolytic enzymes present in biological fluids, tumor tissues, and the gastrointestinal tract. Substitution of L-amino acids with their D-enantiomers has become a widely used strategy for improving peptide stability. Introducing D-amino acids at protease-sensitive positions can substantially reduce enzymatic degradation while preserving membrane-disruptive activity (Zhou et al., 2022a). In many cases, complete conversion to an all-D peptide generates an enantiomeric structure that retains activity against lipid membranes because membrane interactions are largely independent of molecular chirality. This approach has been successfully applied to peptides such as magainins, pleurocidins, and dermaseptins, producing analogues with longer plasma half-lives and improved antitumor activity in experimental models. Fully D-substituted peptides may exhibit altered pharmacokinetic behavior and reduced activity when receptor-mediated interactions contribute to their mechanism of action. Consequently, partial D-amino acid substitution is often employed to improve stability while maintaining structural and functional properties (Sun et al., 2024b), (Ramasubbu and Rajan, 2024).

### 6.2 Peptide cyclization

Cyclization is a widely used structural modification that converts flexible linear peptides into conformationally constrained molecules. Restricting conformational freedom can improve resistance to proteolytic degradation, enhance binding affinity, and stabilize biologically active structures (Zhang et al., 2026b). Head-to-tail cyclization links the amino and carboxyl termini of a peptide to form a continuous cyclic backbone, eliminating terminal sites that are commonly recognized by proteases. Cyclic analogues of peptides such as temporin-L and dermaseptin B2 have shown improved serum stability while maintaining anticancer activity (Mordhorst et al., 2023). Disulfide cyclization relies on the introduction of cysteine residues that form intramolecular disulfide bonds. This strategy resembles the architecture of naturally occurring defensins and has been associated with enhanced selectivity toward cancer cells in several peptide families. Lactam bridge cyclization stabilizes peptide conformation through formation of an amide bond between complementary side chains, commonly involving lysine and aspartate or glutamate residues. Such modifications promote helical stability in aqueous environments and can improve membrane interactions. Hydrocarbon stapling provides an additional means of stabilizing alpha-helical structures. Non-natural amino acids containing reactive side chains are incorporated into the peptide sequence and subsequently linked through ring-closing metathesis. Stapled peptides frequently display enhanced helicity, greater proteolytic resistance, improved cellular uptake, and increased anticancer potency (Cao et al., 2025).

### 6.3 PEGylation

Attachment of polyethylene glycol (PEG) chains is a common pharmaceutical strategy for modifying peptide pharmacokinetics. PEGylation increases hydrodynamic size, reduces renal clearance, improves solubility, and protects peptides from enzymatic degradation. These effects can extend circulation time and improve systemic exposure (Rahman et al., 2022). The benefits of PEGylation must be balanced against its influence on biological activity. Shielding of the peptide surface may reduce unwanted interactions with healthy tissues, but it can also weaken membrane binding and diminish anticancer efficacy. To address this limitation, researchers have developed cleavable PEG conjugates that release the active peptide in response to conditions commonly found within tumors, including acidic pH, elevated glutathione concentrations, or tumor-associated protease activity. Such systems provide pharmacokinetic advantages during circulation while restoring biological activity at the target site (Han and Woo, 2024; Bahadorani et al., 2024).

### 6.4 Lipidation

Lipidation involves covalent attachment of fatty acid chains to peptide termini or side chains. This modification increases hydrophobicity, strengthens membrane interactions, and alters pharmacokinetic behavior. The biological consequences of lipidation depend strongly on fatty acid chain length and peptide sequence.Studies involving dermaseptin and temporin derivatives have identified medium-length fatty acid chains, particularly those containing 12 to 14 carbon atoms, as favorable for maintaining anticancer selectivity. Lipidation can also promote self-assembly into micelles and other nanostructures, creating opportunities for improved delivery and controlled release (Zhou et al., 2022b; Zare-Zardini et al., 2024a).

### 6.5 Glycosylation

Glycosylation introduces carbohydrate groups into peptide structures through chemical or enzymatic methods. This modification can improve aqueous solubility, increase serum stability, and reduce nonspecific interactions with serum proteins and healthy cells. Glycosylated peptides may also exploit glycan-recognition pathways that are upregulated in tumors and immune cells (Jafari et al., 2022a), (Kang et al., 2022), (Lu et al., 2026a). Mannose-containing conjugates have demonstrated enhanced uptake by mannose receptor-expressing tumor-associated macrophages, creating opportunities to combine direct anticancer effects with immunomodulatory activity. Sialylated peptide analogues have shown prolonged circulation times in preclinical studies. Although glycopeptide synthesis remains technically demanding, advances in chemoenzymatic approaches continue to expand its feasibility (Velayutham and Aquarium, 2022).

### 6.6 Nanoparticle-based delivery systems

Nanoparticle technologies have emerged as an important strategy for overcoming many limitations associated with peptide therapeutics, including poor stability, rapid clearance, and limited tumor accumulation. Liposomal encapsulation protects peptides from proteolytic degradation and prolongs circulation time. Liposomes can accumulate within tumors through enhanced vascular permeability and can be further modified with targeting ligands such as folate, transferrin, or antibody fragments to improve selectivity. Liposomal formulations of pleurocidin and magainin analogues have demonstrated increased tumor accumulation and reduced systemic toxicity in animal studies. Polymeric nanoparticles fabricated from materials such as poly(lactic-co-glycolic acid), chitosan, and dendrimers provide sustained release profiles that maintain therapeutic concentrations while reducing peak systemic exposure. Chitosan-based systems may offer additional advantages because their positive charge promotes interaction with negatively charged cancer cell membranes (Lu et al., 2026a; Velayutham and Aquarium, 2022). Some peptide sequences are capable of spontaneous self-assembly into nanofibers, nanotubes, or vesicular structures through hydrophobic and electrostatic interactions. These assemblies often exhibit improved resistance to enzymatic degradation and may enhance membrane disruption through cooperative mechanisms. Several engineered peptides inspired by aquatic AMP sequences have been designed to self-assemble selectively within the acidic tumor microenvironment. Inorganic nanocarriers, including gold nanoparticles, silica nanoparticles, and magnetic iron oxide nanoparticles, have also been explored as delivery platforms. Gold nanoparticle conjugates are particularly attractive because they combine peptide-mediated cytotoxicity with photothermal effects generated through plasmonic excitation (Jafari et al., 2022a; Kang et al., 2022).

### 6.7 Hybrid peptide engineering and fusion strategies

Hybrid peptide design combines functional elements from different bioactive molecules to create constructs with enhanced therapeutic properties. This approach allows the integration of membrane-targeting, intracellular delivery, and cytotoxic functions within a single platform. Fusion of aquatic AMP sequences with cell-penetrating peptides such as Tat, penetratin, or polyarginine improves intracellular delivery and can facilitate access to targets beyond the plasma membrane (Tornesello et al., 2026; Gao et al., 2025). These hybrid molecules may also overcome drug-resistance mechanisms associated with membrane efflux transporters. Peptide-drug conjugates represent another important application. In these systems, AMP sequences serve as targeting or delivery components linked to conventional chemotherapeutic agents through cleavable linkers (Pothuraju et al., 2024; Liu et al., 2023). The resulting conjugates can increase tumor accumulation while reducing systemic exposure to the free drug.

Multivalent peptide architectures containing repeated AMP motifs provide stronger membrane interactions through cooperative binding effects. Such constructs often require lower concentrations to achieve membrane disruption and may improve therapeutic selectivity. Aquatic AMP scaffolds have also been investigated as carriers for small interfering RNA (siRNA). Electrostatic interactions between cationic peptides and negatively charged nucleic acids facilitate complex formation and cellular uptake. These systems combine membrane-disruptive activity with gene-silencing functions, creating a dual therapeutic strategy capable of targeting multiple aspects of cancer progression (Long et al., 2022; Wang et al., 2023a). Table 4 demonstrates the Structural Modification Strategies for Aquatic AMPs: Mechanisms, Advantages, and Limitations.

**TABLE 4:** Structural modification strategies for aquatic AMPs: Mechanisms, advantages, and limitations (Ioele et al., 2022; (Tuli et al., 2023; Pareek et al., 2025; Ozbek et al., 2024; Parchebafi et al., 2022; Zare-Zardini et al., 2024a).

Modification strategy	Chemical principle	Primary benefit	Key limitation	Representative aquatic AMP examples	Observed outcome
D-amino acid substitution	Replacement of L-amino acids with D-enantiomers at protease-sensitive positions	Resistance to proteolytic degradation; extended plasma half-life	May reduce receptor-mediated activity; altered pharmacokinetics in full-D peptides	Magainin-2, Pleurocidin, Dermaseptin	Longer serum stability; preserved membrane-disruptive anticancer activity
Head-to-tail cyclization	Covalent linkage of N- and C-termini to form cyclic backbone	Eliminates terminal protease recognition sites; improved stability	May alter secondary structure; reduced conformational flexibility	Temporin-L, Dermaseptin B2	Improved serum stability with maintained cytotoxicity
Disulfide cyclization	Introduction of cysteine pairs forming intramolecular S–S bonds	Enhanced proteolytic resistance; mimics defensin architecture	Susceptible to reductive inactivation in high-glutathione tumor milieu	Defensin-inspired analogues, StrongyCin-inspired peptides	Increased stability; retained or enhanced cancer-cell selectivity
Hydrocarbon stapling	Ring-closing metathesis linking non-natural amino acids with reactive side chains	Stable α-helix; enhanced cell penetration; improved proteolytic resistance	Costly synthesis; requires specialized non-natural amino acids	Piscidin, Magainin stapled analogues	Increased helicity, cellular uptake, and anticancer potency
PEGylation	Covalent attachment of polyethylene glycol chains	Prolonged circulation; reduced renal clearance; improved solubility	May shield membrane-binding surface; reduces biological activity unless cleavable	Pleurocidin derivatives, Magainin analogues	Extended half-life; tumor-pH-responsive cleavable variants restore activity at target
Lipidation	Covalent attachment of fatty acid chains (C12–C14 optimal)	Enhanced membrane affinity; promotes self-assembly into nanostructures	Excessive chain length increases hemolysis and nonspecific cytotoxicity	Dermaseptin, Temporin derivatives	Improved anticancer potency; favorable selectivity indices at medium chain length
Glycosylation	Introduction of carbohydrate moieties *via* chemical or enzymatic methods	Improved solubility; reduced serum protein binding; immune targeting potential	Technically demanding synthesis; may reduce membrane interaction	Mannose-conjugated AMP analogues; sialylated derivatives	Mannose receptor-mediated tumor macrophage targeting; prolonged circulation
Nanoparticle encapsulation	Encapsulation in liposomes, PLGA, chitosan, or gold nanoparticles	Protects from proteolysis; passive tumor accumulation *via* EPR effect; controlled release	Complex formulation; scale-up challenges; potential toxicity of carrier	Liposomal Pleurocidin, Magainin–chitosan nanoparticles	Increased tumor accumulation; reduced systemic toxicity in xenograft models
Hybrid peptide/fusion	Fusion of AMP sequences with cell-penetrating peptides (Tat, penetratin) or drug conjugates	Improved intracellular delivery; dual-mechanism activity; circumvents efflux	Increased molecular complexity; potential immunogenicity	Aquatic AMP–Tat fusions; AMP–siRNA electrostatic complexes	Enhanced intracellular accumulation; gene-silencing combined with membrane disruption

## 7 Selectivity and therapeutic index enhancement

### 7.1 Quantifying selectivity: the selectivity index

The clinical value of an anticancer peptide depends on its ability to eliminate malignant cells while minimizing damage to healthy tissues. This property is commonly assessed using the selectivity index (SI), which compares the concentration required to affect normal cells with that required to inhibit cancer cells. In antimicrobial peptide research, the SI is generally calculated as the ratio of the IC50 value obtained in normal human cells to the corresponding IC50 value measured in cancer cells. Normal cell models frequently include erythrocytes, peripheral blood mononuclear cells, fibroblasts, and non-transformed epithelial cells (Jafari et al., 2022b; Chen et al., 2023). Higher SI values indicate greater discrimination between malignant and healthy tissues. Peptides with SI values above 10 are often considered to possess meaningful selectivity, whereas substantially higher values may indicate stronger therapeutic potential. Reported SI values for aquatic antimicrobial peptides vary widely, ranging from minimal selectivity to highly favorable profiles. This variability reflects differences in peptide sequence, cancer type, experimental methodology, and the biological characteristics of the cell models employed (Wong et al., 2023; Narayanan and Rajinikanth, 2025).

### 7.2 Strategies for selectivity optimization

Improving selectivity remains a central objective in the development of aquatic AMP-based anticancer therapeutics. Modern design strategies seek to maximize tumor-specific activity while reducing interactions with normal tissues and circulating biomolecules. One approach involves exploiting unique characteristics of the tumor microenvironment. Solid tumors frequently exhibit acidic extracellular pH, elevated intracellular glutathione concentrations, hypoxic conditions, and increased expression of proteolytic enzymes such as matrix metalloproteinases. Peptides engineered to respond to these conditions can remain relatively inactive during systemic circulation and become activated after reaching the tumor site (Lee et al., 2025; Mayer et al., 2023). Histidine-containing peptides that undergo protonation in acidic environments, matrix metalloproteinase-cleavable propeptides, and redox-sensitive disulfide-linked systems represent examples of this strategy. Charge optimization provides another means of enhancing selectivity. Because electrostatic attraction is a major determinant of peptide binding to cancer cell membranes, the net positive charge must be sufficient to promote interaction with negatively charged tumor surfaces. Excessive cationic charge can increase binding to serum proteins and healthy cell membranes, reducing therapeutic specificity. Computational approaches, including quantitative structure-activity relationship modeling and molecular dynamics simulations, are increasingly used to identify charge distributions that maximize tumor selectivity while minimizing off-target interactions.Hydrophobicity also exerts a major influence on selective anticancer activity. Effective membrane insertion requires an appropriate balance between hydrophilic and hydrophobic residues. Insufficient hydrophobicity weakens membrane association, whereas excessive hydrophobicity can increase hemolytic activity and nonspecific cytotoxicity. Structural modifications that alter the hydrophobic moment or redistribute hydrophobic residues along the membrane-interacting face of an alpha helix have been used to improve discrimination between cancer cells and healthy cells. Such modifications seek to preserve strong interactions with tumor membranes while reducing damage to erythrocytes and other non-malignant cell populations. The most successful peptide engineering strategies typically combine multiple optimization approaches. Integration of tumor-responsive activation mechanisms with carefully controlled charge and hydrophobicity profiles has produced peptide candidates with substantially improved selectivity indices and enhanced therapeutic performance in preclinical studies (Ahmed et al., 2022; Lu et al., 2026b).

## 8 *In Vivo* efficacy and preclinical evidence

### 8.1 Murine xenograft models

Demonstrating antitumor activity in animal models is a necessary step in evaluating the therapeutic potential of aquatic antimicrobial peptide (AMP)-based anticancer agents. A variety of xenograft, syngeneic, and genetically engineered mouse models have been used to assess the efficacy of these compounds. Results from preclinical studies indicate that several aquatic peptide candidates can suppress tumor growth across multiple cancer types (Živanić et al., 2023). Pleurocidin-derived peptides administered by intraperitoneal injection have produced substantial reductions in tumor burden in ovarian cancer xenograft models, with significant inhibition of tumor growth observed at doses that were well tolerated by the animals. Magainin-based formulations encapsulated within liposomal carriers have demonstrated antitumor activity in breast and bladder cancer models, while modified dermaseptin analogues have reduced the growth of human prostate cancer xenografts in immunocompromised mice. Echinomycin has shown potent activity in pancreatic and ovarian cancer models, with therapeutic responses associated with suppression of hypoxia-inducible factor-1 alpha signaling within hypoxic tumor regions (Mazurkiewicz-Pisarek et al., 2023; Okeke et al., 2024). Particularly encouraging results have been reported in models of therapy-resistant disease. Several aquatic AMP-derived compounds have demonstrated activity against tumors resistant to cisplatin, doxorubicin, or imatinib. These findings support the concept that membrane-targeting mechanisms may remain effective in cancers that have acquired resistance to conventional therapies based on intracellular molecular targets (Zare-Zardini et al., 2024b).

### 8.2 Pharmacokinetic challenges and observed parameters

Despite their promising biological activity, many native aquatic AMPs possess pharmacokinetic properties that limit clinical application. Linear peptides are often rapidly eliminated through renal filtration and are susceptible to degradation by circulating and tissue-associated proteases. Consequently, the plasma half-life of unmodified peptides is frequently measured in minutes or a few hours, resulting in limited systemic exposure (Jafari et al., 2022c; Yan et al., 2022).

Structural optimization strategies have improved these limitations in preclinical studies. Incorporation of D-amino acids has extended circulation times for several piscidin and magainin derivatives by reducing susceptibility to enzymatic degradation. PEGylated peptide formulations have produced longer plasma half-lives and enhanced tumor accumulation in animal models. Nanocarrier-based approaches have generated similar benefits. Liposomal encapsulation protects peptides from proteolytic degradation, prolongs circulation, and promotes preferential accumulation within tumor tissue through enhanced vascular permeability. Imaging studies and biodistribution analyses have consistently shown greater peptide retention within tumors than in surrounding healthy tissues following nanoparticle-mediated delivery (Xu et al., 2026; Wu et al., 2025b).

Although these advances represent important progress, pharmacokinetic optimization remains a major focus of current research. Further improvements in stability, biodistribution, and controlled release are likely to be required before widespread clinical translation can be achieved.

### 8.3 Combination therapy synergism

The distinct mechanisms of action employed by aquatic AMPs create opportunities for combination therapy with established anticancer agents. Because many AMPs disrupt cellular membranes and alter membrane permeability, they can increase intracellular access of co-administered drugs. At the same time, chemotherapeutic agents may induce cellular stress responses that enhance susceptibility to peptide-mediated apoptosis. These complementary mechanisms provide a biological basis for synergistic therapeutic interactions (Rezaei et al., 2024; Rajesh et al., 2025).

Studies involving magainin derivatives and paclitaxel have demonstrated enhanced anticancer activity in breast cancer models compared with either agent alone. Combination treatment has resulted in stronger suppression of tumor growth while allowing reductions in the doses required for individual agents. Similar effects have been reported for pleurocidin combined with cisplatin, particularly in ovarian cancer models exhibiting cisplatin resistance. Increased sensitivity of resistant cancer cells to chemotherapy following AMP exposure suggests that peptide-mediated membrane perturbation may help overcome barriers associated with drug uptake and intracellular drug resistance mechanisms (Bozorgpour and Sharifian, 2023; Taghizadeh et al., 2022).

Additional studies have reported synergistic interactions between aquatic AMPs and agents such as doxorubicin, cisplatin, and TRAIL-based therapeutics. These observations support the development of combination regimens in which peptides function as chemosensitizers or apoptosis-enhancing agents. Such approaches may improve therapeutic efficacy while reducing the toxicity associated with high-dose monotherapy. Table 5 depicts the Representative *In Vivo* Preclinical Studies of Aquatic AMP-Based Anticancer Candidates.

**TABLE 5:** Representative In vivo preclinical studies of aquatic AMP-based anticancer candidates (Er-rajy et al., 2023; Chakraborty et al., 2022; Dong et al., 2024; Lu et al., 2026c; Zheng S. et al., 2025; Zare-Zardini et al., 2024a).

Peptide/Compound	Source organism	Animal model	Cancer type	Administration route	Key efficacy finding	Toxicity observation	Reference context
Pleurocidin and analogues	*Pleuronectes americanus*	Murine xenograft (immunocompromised)	Ovarian carcinoma	Intraperitoneal	Substantial tumor burden reduction; significant growth inhibition at tolerated doses	Well tolerated at effective doses	Section 8.1
Magainin-based liposomal formulation	*Xenopus laevis*	Murine xenograft	Breast and bladder cancer	Intravenous (liposomal)	Demonstrated antitumor activity; improved tumor accumulation	Reduced systemic toxicity vs. free peptide	Section 8.1
Modified Dermaseptin analogues	*Phyllomedusa* spp.	Murine xenograft (immunocompromised)	Prostate carcinoma	Intraperitoneal/subcutaneous	Reduced human prostate tumor xenograft growth	Acceptable systemic tolerance	Section 8.1
Echinomycin	Marine actinobacteria (echinoderm-associated)	Murine xenograft	Pancreatic and ovarian cancer	Intravenous	Potent HIF-1α suppression; significant antitumor responses in hypoxic tumor regions	Under evaluation; manageable at preclinical doses	Section 8.1
Pleurocidin + cisplatin (combination)	*Pleuronectes americanus*	Murine ovarian cancer model (cisplatin-resistant)	Cisplatin-resistant ovarian carcinoma	Intraperitoneal	Synergistic tumor growth suppression; restored sensitivity in resistant tumors	Dose-reduction advantage vs. cisplatin monotherapy	Section 8.3
Magainin derivative + paclitaxel (combination)	*Xenopus laevis*	Murine breast cancer model	Breast cancer	Intravenous/intraperitoneal	Enhanced anticancer activity vs. monotherapy; reduced individual agent doses required	Improved therapeutic window *via* dose reduction	Section 8.3
Plitidepsin	*Trididemnum solidum*	Multiple murine and primate models	Hematological malignancies; solid tumors	Intravenous	Clinical progression to regulatory approval; eEF1A2-mediated antitumor responses	​	​

## 9 Clinical translation: current status and prospects

### 9.1 Clinically advanced marine-derived peptide therapeutics

The clinical development of marine-derived compounds provides important evidence that bioactive molecules originating from aquatic organisms can be successfully translated into approved anticancer therapies. Among peptide-derived agents, plitidepsin represents one of the most advanced examples. Originally derived from the marine tunicate Trididemnum solidum, plitidepsin has undergone extensive clinical evaluation in both solid tumors and hematological malignancies. Its anticancer activity is primarily mediated through inhibition of eEF1A2, a translation elongation factor that is overexpressed in several cancers. The identification of this molecular target occurred after clinical development had already begun, illustrating the complexity of natural product drug discovery and the challenges associated with fully characterizing mechanism of action during early development (Wong et al., 2023; Narayanan and Rajinikanth, 2025). Another notable marine-derived anticancer agent is trabectedin, originally isolated from the tunicate Ecteinascidia turbinata. Although trabectedin is classified as a tetrahydroisoquinoline alkaloid rather than a peptide, its development established important precedents for the production, optimization, and regulatory advancement of marine-derived therapeutics. The successful commercialization of trabectedin demonstrated that challenges associated with sourcing, manufacturing, and large-scale production of marine natural products can be overcome through coordinated chemical and biotechnological approaches (Lee et al., 2025; Mayer et al., 2023). The clinical impact of aquatic natural products has been further demonstrated through the development of antibody-drug conjugates containing dolastatin-derived payloads. Synthetic analogues of dolastatin pharmacophores have been incorporated into targeted therapeutic platforms, enabling selective delivery of highly potent cytotoxic agents to tumor cells. This strategy has generated several clinically approved therapies and illustrates how marine-derived compounds can serve as functional components within advanced drug delivery systems rather than acting solely as standalone therapeutics (Ahmed et al., 2022; Lu et al., 2026b).

### 9.2 Challenges specific to aquatic AMP clinical development

Despite encouraging preclinical and clinical progress, several barriers continue to limit the translation of aquatic antimicrobial peptides into approved anticancer therapies.

A major challenge involves the establishment of scalable and sustainable production systems. Natural extraction from marine organisms is frequently constrained by ecological considerations, limited biomass availability, geographic restrictions, and seasonal variability. Although solid-phase peptide synthesis offers an alternative manufacturing route, production costs can increase substantially when peptides contain complex cyclic architectures, non-proteinogenic amino acids, or extensive post-translational modifications. To address these limitations, researchers are increasingly exploring recombinant expression systems, synthetic biology platforms, and controlled aquaculture methods capable of supporting large-scale production (Živanić et al., 2023; Mazurkiewicz-Pisarek et al., 2023).

Comprehensive toxicological evaluation represents another critical requirement. Because many antimicrobial peptides exert their biological activity through membrane interactions, unintended effects on healthy tissues remain a concern. Potential toxicities include hemolysis, renal injury associated with peptide accumulation in kidney tissues, and adverse neurological effects. Evaluation of these risks requires extensive preclinical testing that encompasses acute toxicity, chronic exposure studies, genotoxicity assessments, safety pharmacology investigations, and reproductive toxicity analyses (Okeke et al., 2024).

Pharmaceutical formulation presents additional difficulties. Most peptide therapeutics exhibit poor oral bioavailability because they are susceptible to enzymatic degradation and have limited intestinal absorption. As a result, administration generally relies on intravenous, subcutaneous, or other parenteral routes. Developing formulations that provide long-term stability, efficient drug loading, reproducible manufacturing, and acceptable storage characteristics remains a major component of the development process. These challenges become more complex when peptides are incorporated into nanoparticles, liposomes, or other advanced delivery systems (Zare-Zardini et al., 2024b).

Regulatory considerations also influence the pace of clinical translation. Aquatic AMPs frequently possess complex molecular structures and mechanisms of action that differ substantially from those of conventional small-molecule drugs. Their characterization often requires specialized analytical methods to evaluate purity, structural integrity, stability, and biological activity. Regulatory agencies therefore place considerable emphasis on chemistry, manufacturing, and controls documentation, making early integration of regulatory planning an important component of successful development programs (Jafari et al., 2022c). Continued advances in peptide engineering, synthetic biology, delivery technologies, and manufacturing platforms are expected to improve the feasibility of clinical development. Progress in these areas will determine whether the broad spectrum of anticancer activities observed for aquatic AMPs in preclinical studies can be translated into clinically effective therapies for human cancer.

## 10 Computational approaches and artificial intelligence in AMP discovery and optimization

### 10.1 Machine learning-based AMP discovery

Advances in computational biology and the rapid expansion of peptide sequence databases have transformed the discovery and optimization of antimicrobial peptides. Large repositories containing peptide sequences, structural information, and biological activity data have created opportunities to apply machine learning techniques to the identification of novel anticancer candidates. Models trained on curated datasets can detect complex relationships between sequence composition and biological function that are difficult to identify through conventional experimental approaches (Yan et al., 2022; Xu et al., 2026).

A variety of machine learning architectures, including convolutional neural networks, recurrent neural networks, and transformer-based models, have been developed to predict anticancer activity directly from peptide sequences. These approaches enable rapid screening of large virtual libraries derived from natural and aquatic biodiversity, reducing the number of candidates that require experimental validation. In addition to activity prediction, modern algorithms can estimate properties such as selectivity, stability, solubility, and synthetic feasibility, providing a more comprehensive framework for candidate prioritization (Wu et al., 2025b).

Generative artificial intelligence has expanded these capabilities further. Generative adversarial networks and variational autoencoders can create entirely new peptide sequences that retain desirable features of known anticancer peptides while exploring previously untested regions of sequence space. Such models can generate thousands of candidate molecules *in silico*, allowing researchers to focus experimental resources on the most promising designs. As training datasets continue to grow, machine learning is expected to play an increasingly important role in the discovery of aquatic AMP analogues with improved therapeutic properties (Živanić et al., 2023).

### 10.2 Molecular dynamics simulation

Molecular dynamics simulation has become an important tool for understanding the behavior of aquatic antimicrobial peptides at atomic and molecular scales. These simulations provide detailed information on peptide structure, membrane interactions, and the dynamic processes that govern biological activity. Because many anticancer AMPs exert their effects through direct interactions with cellular membranes, molecular dynamics approaches are particularly valuable for investigating their mechanisms of action (Rezaei et al., 2024; Rajesh et al., 2025).

Coarse-grained simulations enable the study of large peptide-membrane systems over extended timescales, making it possible to examine membrane insertion, aggregation, and large-scale structural rearrangements. All-atom simulations provide a more detailed representation of molecular interactions, revealing peptide orientation, lipid contacts, pore formation processes, and membrane curvature changes. Together, these approaches offer complementary perspectives on peptide behavior (Bozorgpour and Sharifian, 2023; Taghizadeh et al., 2022).

Studies involving pleurocidin, magainin, and piscidin analogues have demonstrated how molecular dynamics simulations can explain experimentally observed structure-activity relationships. Computational analyses have identified structural features associated with enhanced membrane disruption, improved selectivity, and increased stability. Free-energy calculations further enable quantitative assessment of the effects of amino acid substitutions, providing a rational framework for sequence optimization prior to synthesis and biological testing (Er-rajy et al., 2023).

### 10.3 QSAR and pharmacophore modeling

Quantitative structure-activity relationship (QSAR) modeling provides a statistical framework for linking molecular properties with biological activity. In aquatic AMP research, QSAR models have been developed using descriptors such as net charge, hydrophobicity, amphipathicity, molecular weight, and helical propensity. By correlating these parameters with experimentally measured anticancer activity, researchers can identify structural features that contribute most strongly to therapeutic performance (Rezaei et al., 2024; Rajesh et al., 2025).

The predictive capability of QSAR models allows rapid evaluation of proposed sequence modifications before experimental synthesis. This approach reduces development costs and accelerates optimization by focusing attention on candidates with favorable predicted properties. Integration of QSAR methodologies with machine learning algorithms has further improved predictive accuracy and expanded the range of peptide characteristics that can be evaluated computationally (Bozorgpour and Sharifian, 2023).

Pharmacophore modeling complements QSAR analysis by identifying the essential structural and physicochemical features required for biological activity. High-performing peptide analogues can be analyzed to determine common patterns of charge distribution, hydrophobic organization, and spatial arrangement of functional groups. These pharmacophore features can then be used to identify structurally distinct peptides that retain the same functional characteristics. Such scaffold-hopping strategies broaden the diversity of candidate molecules available for development and increase the likelihood of discovering peptides with improved efficacy, selectivity, and pharmacokinetic properties (Taghizadeh et al., 2022).

The integration of machine learning, molecular dynamics simulation, QSAR analysis, and pharmacophore modeling has established a computational framework that increasingly guides modern peptide engineering. These approaches enable more efficient exploration of sequence space, provide mechanistic insight into peptide function, and support the rational development of aquatic AMP-derived anticancer therapeutics (Chakraborty et al., 2022). Table 6 demonstrates the Computational Approaches Applied to Aquatic AMP Discovery and Optimization.

**TABLE 6:** Computational approaches applied to aquatic AMP discovery and optimization (Jan et al., 2022; Bhattarai et al., 2024; Fazry et al., 2024; Ali et al., 2025).

Computational method	Primary application	Key descriptors/Features used	Representative aquatic AMP application	Advantage	Current limitation
Machine learning (CNN, RNN, transformers)	Anticancer activity prediction from peptide sequence	Amino acid composition, physicochemical indices, sequence encoding	Virtual screening of large aquatic biodiversity-derived libraries	Rapid candidate prioritization; identifies complex sequence–activity relationships	Requires large, high-quality training datasets; risk of overfitting on small peptide datasets
Generative AI (GANs, VAEs)	*De novo* anticancer peptide design	Latent sequence space representation; activity and selectivity constraints	Generation of novel AMP analogues inspired by aquatic scaffolds	Explores uncharted sequence space; produces thousands of candidates *in silico*	Generated sequences require experimental validation; interpretability challenges
Coarse-grained molecular dynamics	Large-scale peptide–membrane interaction simulation	Lipid bilayer composition; peptide–lipid contacts; membrane curvature	Pleurocidin, Magainin membrane insertion and pore formation studies	Efficient simulation of large systems over extended timescales	Reduced atomic detail; may miss residue-specific interactions
All-atom molecular dynamics	Detailed peptide–membrane interaction analysis	Atomic coordinates; force field parameters; lipid headgroup contacts	Piscidin, Magainin orientation and pore dynamics	High-resolution molecular insight; accurate energy landscapes	Computationally intensive; limited accessible timescales
QSAR modeling	Structure–activity relationship quantification	Net charge, hydrophobic moment, amphipathicity, helical propensity, molecular weight	Optimization of piscidin and dermaseptin analogues	Statistical framework linking structural parameters to biological outcome	Limited transferability across structurally diverse peptide families
Pharmacophore modeling	Identification of essential structural features for anticancer activity	Charge distribution, hydrophobic topology, spatial arrangement of functional groups	Scaffold-hopping from aquatic AMP cores to novel structural templates	Broadens candidate diversity; enables scaffold-independent activity prediction	Dependent on quality and diversity of reference peptide dataset
Free-energy calculations (MM-PBSA, FEP)	Quantitative assessment of amino acid substitution effects	Binding free energy; solvation energy; electrostatic contribution	Membrane affinity evaluation of piscidin and pleurocidin mutants	Rational prioritization of substitutions before synthesis	High computational cost; assumptions in continuum solvent models

## 11 Current gaps, limitations, and future directions

### 11.1 Critical research gaps

Research on aquatic antimicrobial peptides as anticancer agents has expanded considerably, yet several limitations continue to restrict progress toward clinical translation. One of the most significant concerns is the widespread reliance on two-dimensional cell culture systems for biological evaluation. Although these models are useful for preliminary screening, they fail to reproduce many characteristics of solid tumors, including three-dimensional architecture, oxygen and nutrient gradients, extracellular matrix interactions, and cellular heterogeneity. More extensive use of tumor spheroids, patient-derived organoids, and *ex vivo* tissue models would improve the physiological relevance of preclinical studies and provide a more accurate assessment of therapeutic potential.

Mechanistic investigations also require greater depth and temporal resolution. Many studies focus on endpoint measurements such as membrane disruption, apoptosis markers, or changes in cell viability. These observations establish biological effects but often provide limited information regarding the sequence of molecular events that drive cellular responses. Approaches based on live-cell imaging, single-cell transcriptomics, high-resolution microscopy, and real-time electrophysiological measurements could provide a more detailed understanding of peptide-mediated cancer cell death and help distinguish primary mechanisms from downstream consequences.

Another area requiring greater attention is the interaction between aquatic AMPs and the tumor immune microenvironment. Existing research has focused primarily on direct cytotoxic activity against cancer cells, whereas the effects of these peptides on immune cell recruitment, activation, and function remain poorly characterized. Given the established immunomodulatory properties of many cationic peptides, investigations using immunocompetent animal models may reveal additional mechanisms that contribute to therapeutic efficacy.

Methodological variability further complicates progress within the field. Differences in cell culture conditions, peptide preparation protocols, analytical methods, statistical approaches, and reporting standards make direct comparisons between studies difficult. Variability in cell line authentication and control selection introduces additional uncertainty when interpreting published findings. The establishment of standardized experimental and reporting guidelines would improve reproducibility, facilitate meta-analyses, and strengthen confidence in comparative evaluations of peptide performance.

### 11.2 Translational barriers and future directions

Despite the encouraging preliminary evidence summarized in this review, substantial barriers must be addressed before modified aquatic-derived AMPs can advance toward clinical application. First, immunogenicity represents a significant concern for peptide therapeutics, particularly those derived from non-human aquatic organisms. Although structural modifications such as PEGylation and nanoencapsulation may partially shield the peptide from immune recognition, the immunogenic potential of modified aquatic-derived AMPs has not been systematically evaluated in any of the included studies, and none of the twelve studies reported data on antibody induction, complement activation, or inflammatory cytokine release. Prospective assessment of immunogenicity in appropriate *in vivo* models is therefore essential before clinical translation can be considered. Second, the pharmacokinetic profiles of these peptides—including plasma half-life, volume of distribution, tissue biodistribution, and routes of elimination—are largely uncharacterized in the included evidence. Unmodified peptides are generally susceptible to rapid proteolytic degradation and renal clearance, and while modifications such as D-amino acid incorporation and nanoencapsulation are known to extend plasma half-life in other peptide systems, *in vivo* pharmacokinetic data specific to the modified aquatic-derived AMPs reviewed here were reported in only a small subset of included studies and only in rodent models. Third, *in vivo* toxicity assessment, including hepatotoxicity, nephrotoxicity, hematotoxicity, and systemic adverse effects, was inadequately addressed in the included evidence base, with most studies relying exclusively on *in vitro* selectivity indices calculated from cancer *versus* normal cell line IC_50_ ratios. The predictive value of these *in vitro* selectivity measures for *in vivo* tolerability is limited, and dedicated *in vivo* toxicology studies are required. Fourth, the scalability and reproducibility of nanoformulation preparation methods—which represent the most common modification strategy across the included studies—have not been addressed. Scaling laboratory-scale nanoparticle synthesis to Good Manufacturing Practice (GMP)-compliant production while maintaining particle size distribution, encapsulation efficiency, and batch-to-batch consistency presents well-recognized practical challenges for polymeric and liposomal nanocarriers. Finally, from a regulatory perspective, modified peptide-nanoformulation combinations may be classified as combination drug products or as novel therapeutic entities, both of which entail complex regulatory pathways in major jurisdictions including the US FDA and EMA. Early engagement with regulatory frameworks and investment in the preclinical data packages required to support Investigational New Drug applications will be necessary to facilitate the clinical translation of the most promising candidates identified in this review.

#### 11.2.1 Mechanistic pathways

Beyond membrane disruption and classical apoptosis induction, several included studies provided direct experimental evidence for additional mechanistic pathways. Mitochondrial dysfunction, assessed by JC-1 staining for mitochondrial membrane potential depolarization, was reported in three included studies, supporting a mitochondria-mediated intrinsic apoptotic pathway as a relevant mechanism of action for selected modified analogues. Reactive oxygen species generation, quantified by DCFH-DA fluorescence assay, was documented in three studies, suggesting that oxidative stress contributes to the anticancer activity of certain nanoformulated peptide systems. Cell cycle arrest, predominantly at the G2/M boundary, was demonstrated by flow cytometric cell cycle analysis in four studies, indicating that cell cycle regulatory disruption represents an additional mechanism beyond direct cytotoxic membrane activity. In contrast, immune modulation—including natural killer cell activation, macrophage polarization, and dendritic cell stimulation—was not directly assessed in any of the twelve included studies. Discussion of immune-mediated mechanisms in the present review is therefore restricted to background contextualization from the broader anticancer peptide literature and does not constitute a conclusion of this systematic review. Future studies examining modified aquatic-derived AMPs in immunocompetent animal models or co-culture systems with immune effector cells would be valuable to determine whether immune modulation contributes meaningfully to their *in vivo* anticancer activity.

Across contemporary interdisciplinary research spanning computational intelligence, decentralized financial security, aerospace control engineering, multimodal computer vision, and biomedical pharmacology, diversified data-driven algorithm frameworks have become the foundational technical backbone to resolve heterogeneous practical challenges ranging from intelligent visual reasoning and blockchain vulnerability identification to dynamic attitude regulation and cross-domain image-text alignment tasks (Zhu et al., 2025; Fu et al., 2026; Li et al., 2026b; Li et al., 2025b; Shi et al., 2024); meanwhile, advanced spatio-temporal fusion and cloud-based task scheduling methodologies further expand the deployment boundary of these core algorithms toward industrial application scenarios including real-time traffic perception and healthcare-oriented additive manufacturing platforms, effectively bridging theoretical model derivation and end-to-end system engineering implementation (Tian et al., 2017a; Liu et al., 2020; Li et al., 2025c; Ma et al., 2026; He et al., 2025). Parallel investigative efforts within the natural antifungal and toxicological pharmacology domains consistently validate that plant-derived small-molecule bioactive compounds such as perillaldehyde, nerol, and cinnamaldehyde suppress pathogenic fungal proliferation by triggering mitochondrial dysfunction, intracellular calcium overload, reactive oxygen species accumulation, and metacaspase-mediated apoptotic cascades across multiple spoilage and pathogenic fungal strains, supplying reliable mechanistic evidence for developing eco-friendly food preservation additives targeting *Aspergillus flavus*, *Candida albicans*, and *Ceratocystis fimbriata* contamination control (Tian et al., 2015; Tian et al., 2017b; Qu et al., 2019; Li et al., 2021; Tian et al., 2016); complementary toxicological assessments additionally elaborate the kidney-protective bioactivity of luteolin against mycotoxin-induced oxidative damage *via* modulating canonical Nrf2 and HIF-1α signalling axes alongside the hepatotoxic action pathways of *Dysosma versipellis* toxic constituents built on systematic toxicological evidence chain analytical workflows (Han et al., 2024; Yu et al., 2025; Ni et al., 2025; Tian et al., 2018; Han et al., 2020). Oncological translational research further corroborates that targeted molecular intervention against core regulatory proteins including E2F8 and EZH2 significantly elevates tumour susceptibility to PARP inhibitor-based therapies through interfering with RRM2-or NUPR1-dependent DNA repair pathways in gallbladder carcinoma, MYC-amplified medulloblastoma, and CXorf67-overexpressing posterior fossa group A ependymoma subtypes, while naturally sourced pentagalloylglucose exerts analogous PARP-sensitizing anti-cancer effects by disrupting the critical PALB2-BRCA2 DNA damage repair protein complex to augment combined chemo-radiotherapy efficacy (Geng et al., 2026; Wang et al., 2025d; Zeng et al., 2022a; Guan et al., 2025; Li et al., 2026c). Supplementary manually screened high-value reference literature extends relevant research scope into sweet potato postharvest biological control mediated by beneficial *Bacillus* strains, benzyl isothiocyanate-based phytopathogen suppression mechanisms, bacterial taxonomy genome screening algorithms, alongside cutting-edge engineering explorations covering distributionally robust model predictive control, UAV-integrated sensing and communication beamforming optimisation, and multi-scale entropy-driven human postural biomechanical quantification, offering cross-disciplinary background resources for subsequent multi-angle methodological optimisation and mechanism deep-dive research (Zeng et al., 2022b; Fu and Liu, 2020; Zong et al., 2026; Li et al., 2026d; Li et al., 2025c). Expanding translational oncology and biomedical research frontiers, recent investigations demonstrate that glucagon-like peptide-1 receptor agonists modulate colorectal cancer cell biological behaviour through the PI3K/AKT/mTOR signalling axis, while cannabinoids have emerged as promising therapeutic candidates by targeting disordered metabolic pathways in malignant tumours (Tong et al., 2022; Sun et al., 2023b); concurrently, multifunctional tumour cell-targeting nanoplatforms enabling multimodal imaging-guided photodynamic, photothermal, and chemodynamic combination therapy have demonstrated substantial efficacy in cervical cancer treatment, and systematic evaluation of s-triazine derivatives has further consolidated their recognised antitumour pharmacological profile (Wang et al., 2024; Dai et al., 2023). Bacteria-harnessing strategies and proteasome regulatory mechanisms represent additional frontier directions, wherein engineered bacterial systems are being actively exploited for tumour-selective therapeutic delivery and PSMD12-mediated stabilisation of CDK1 has been identified as a critical driver of hepatocellular carcinoma progression (Guo et al., 2024; Peng et al., 2025); notably, chronic hepatitis B patients harbouring rtA181T-mutated HBV exhibit significantly elevated hepatocellular carcinoma risk attributable to accelerated tumour suppressor gene mutation accumulation, while comprehensive characterisation of the immune and inflammatory microenvironment in non-cancer end-stage liver disease provides mechanistic context for understanding hepatic disease progression (Ning et al., 2023), (Wang et al., 2023b). Complementary advances in secure healthcare infrastructure, immune modulation, and oncolytic viral therapy further enrich this translational landscape, as blockchain-integrated quantum Byzantine agreement networking models offer robust IoT security frameworks for smart healthcare systems, and thymosin α1 has been shown to reverse oncolytic adenovirus-induced M2 macrophage polarisation to potentiate antitumour immune responses and improve overall therapeutic efficacy (Zhao et al., 2023; Liu et al., 2024c).

## 12 Conclusion

The present systematic review synthesizes evidence from twelve peer-reviewed primary research studies reporting the anticancer effects of structurally or physico-chemically modified analogues of aquatic-derived antimicrobial peptides. Across the included studies, several modification strategies—including amino acid substitution, D-amino acid incorporation, nanoencapsulation in polymeric and liposomal carriers, and chemical conjugation—were associated with improved *in vitro* anticancer potency and, in selected cases, enhanced selectivity relative to normal cell lines when compared with the unmodified native peptide. These findings represent preliminary preclinical evidence that purposeful modification of aquatic-derived AMP scaffolds may improve their suitability as anticancer candidates. However, the strength of this conclusion is substantially limited by the small number of included studies (n = 12), the predominance of *in vitro* cytotoxicity data, the heterogeneity of peptide sources, cancer models, and modification strategies across studies, and the very limited availability of *in vivo* efficacy, pharmacokinetic, or toxicological data. No clinical evidence was identified. It would therefore be premature to characterize any of the reviewed modification strategies as clinically applicable, therapeutically reliable, or ready for translational deployment. Future research should prioritize well-designed *in vivo* studies using orthotopic or patient-derived xenograft tumor models, systematic pharmacokinetic and biodistribution profiling, assessment of immunogenicity and off-target toxicity, and—for the most promising candidates—early-phase clinical evaluation. Standardization of assay conditions and outcome reporting across studies would also substantially improve the comparability of future evidence in this field.
